# Cell wall remodeling in a fungal pathogen is required for hyphal growth into microspaces

**DOI:** 10.1128/mbio.01184-25

**Published:** 2025-06-30

**Authors:** Hinata Miki, Melani Mariscal Gomez, Ayaka Itani, Daisuke Yamanaka, Yoshikatsu Sato, Antonio Di Pietro, Norio Takeshita

**Affiliations:** 1 Microbiology Research Center for Sustainability (MiCS), Faculty of Life and Environmental Sciences, Tsukuba Institute for Advanced Research (TIAR), University of Tsukuba 13121, Tsukuba, Japan; 2 Departamento de Genética, Campus de Excelencia Internacional Agroalimentario ceiA3, Universidad de Córdoba 16735, Córdoba, Spain; 3 Laboratory for Immunopharmacology of Microbial Products, School of Pharmacy, Tokyo University of Pharmacy and Life Sciences 13115, Hachioji, Tokyo, Japan; 4 Institute of Transformative Bio-Molecules (WPI-ITbM), Nagoya University 12965, Nagoya, Aichi, Japan; Hebrew University of Jerusalem Robert H Smith Faculty of Agriculture Food and Environment, Rehovot, Israel

**Keywords:** hyphae, cell wall, microspace, *Fusarium*, plasticity, microfluidic device, mitogen-activated protein kinases

## Abstract

Hyphae are filamentous structures that are crucial for the lifestyle of many fungi, allowing rapid colonization of diverse substrates such as dead organic material or living host tissue. One of the key features of fungal hyphae is their remarkable morphogenetic plasticity. During host colonization, phytopathogenic fungi must traverse extremely narrow spaces between plant cells such as the apoplastic space or the plasmodesmata. The mechanisms underlying hyphal plasticity during growth into microspaces are poorly understood. Here, we tested a set of isogenic mutants of *Fusarium oxysporum*, an important plant pathogen that causes devastating vascular wilt diseases on more than a hundred crops, for their ability to cross extremely narrow 1 µm channels, approximately one-quarter the width of hyphae. We found that the conserved Mpk1 cell wall integrity mitogen-activated protein kinase (MAPK) cascade is required for efficient growth of hyphae through the microchannels, whereas the two other MAPK pathways, the Fmk1 invasive growth and the Hog1 hyperosmolarity response cascades, are dispensable for this process. Staining with specific probes showed a reduction in both chitin and glucan content in the cell wall of hyphae growing within the 1 µm channel. We further establish that channel passage in cell wall remodeling mutants is impaired due to their inability to constrict hyphal diameter, and this defect can be rescued by increasing extracellular osmolarity. Both the cell wall integrity mutants impaired in microchannel passage, as well as the invasive growth mutants unable to penetrate cellophane membranes, caused significantly reduced mortality in tomato plants. Our study reveals that two morphogenetic processes essential for plant infection, which are governed by distinct cellular pathways, contribute independently to fungal phytopathogenicity.

**IMPORTANCE:**

This study highlights the critical role of hyphal plasticity and cell wall remodeling in the pathogenicity of *Fusarium oxysporum*, a major plant pathogen affecting over a hundred crops. The ability of fungal hyphae to traverse narrow plant tissue spaces, such as apoplastic gaps and plasmodesmata, is essential for successful host colonization. The research demonstrates that the Mpk1 mitogen-activated protein kinase (MAPK) pathway, responsible for cell wall integrity, is crucial for hyphal growth through microspaces, while other MAPK pathways are not. Staining with specific probes showed a reduction in both chitin and glucan content in the cell wall of hyphae growing within 1 µm width channels. Both the cell wall integrity mutants impaired in microchannel passage, as well as the invasive growth mutants unable to penetrate cellophane membranes, caused significantly reduced mortality in tomato plants. Our study reveals that two morphogenetic processes essential for plant infection, which are governed by distinct cellular pathways, contribute independently to fungal phytopathogenicity.

## INTRODUCTION

Filamentous hyphae are crucial for the fungal lifestyle, as they allow the expansion of these nonmotile organisms into new environments, making fungi excellent nutrient scavengers (1). Hyphae can also penetrate into substrates by a process known as invasive growth (IG) (2). Invasive hyphae allow fungal pathogens to efficiently penetrate and colonize the host to extract nutrients and complete their life cycles. Vascular wilt fungi are a particularly destructive group of plant pathogens that attack almost every crop except cereals and are extremely difficult to control (3). The *Fusarium oxysporum* species complex provokes devastating losses in global agriculture (4), exemplified by the highly aggressive clone known as tropical race 4 that threatens to wipe out the world’s industrial Cavendish banana (*Musa acuminata*) production (5). Hyphae of *F. oxysporum* can chemotropically sense and grow toward plant roots (6), enter them through narrow openings between epidermal cells (7), and grow endophytically in the apoplast of the root cortex by releasing early compatibility effectors that promote establishment within the host (8). During a pathogenic interaction, *F. oxysporum* hyphae eventually cross the endodermis to colonize the xylem vessels, leading to clogging, water stress, and progressive wilting of the plant (9).

Among the best-studied cellular pathways mediating fungal infection of plants are mitogen-activated protein kinase (MAPK) cascades (10). Three broadly conserved MAPK pathways have been characterized in a variety of phytopathogens. The Fmk1 IG pathway is homologous to the yeast filamentation pathway and is universally required for fungal pathogenicity in plants by regulating infection-related development and invasive hyphal growth (11–14). A second MAPK cascade, the Mpk1 cell wall integrity (CWI) pathway, mediates cell wall remodeling during fungal development or in response to physical or chemical insults and is also important for plant infection (15). A third MAPK cascade, the Hog1 pathway, is essential for adaptation to hyperosmotic stress but dispensable for plant infection in most phytopathogens (10).

Like many plant pathogenic fungi, *F. oxysporum* uses hyphae to efficiently invade and colonize the host tissues. During the infection process, these filamentous structures must traverse extremely narrow spaces between plant cells such as the apoplastic space or the plasmodesmata (14). The mechanisms underlying such hyphal plasticity are poorly understood. In a recent study, a microfluidic device was used for live cell imaging of hyphae from different fungal species during growth through extremely narrow channels of approximately 1 µm width, revealing an unexpected trade-off between hyphal plasticity and growth speed (16). However, the genetic and cellular underpinnings of hyphal plasticity during entry and elongation into microspaces, as well as their role in fungal pathogenicity, remain largely unknown. Here, we tested a panel of isogenic mutants of *F. oxysporum* affected in genes related to key processes such as invasive hyphal growth or CWI, for their ability to traverse the microfluidic channels. Our results demonstrate that CWI and remodeling components are essential for hyphal growth into microspaces, whereas key regulators of invasive hyphal growth are dispensable for this process.

## RESULTS

### Hyphal passage through narrow channels does not depend on the conserved invasive growth and hyperosmolarity response MAPK pathways

**Fig 1:**
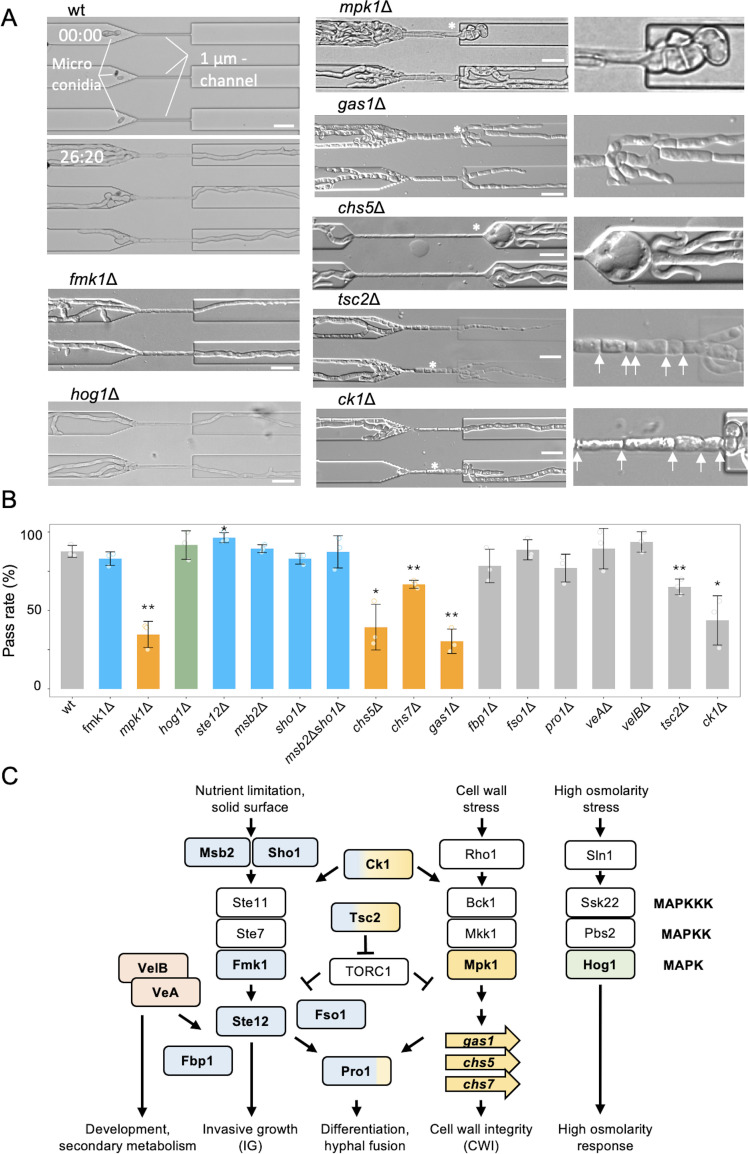
CWI mutants are impaired in the hyphal growth of *F. oxysporum* across microchannels. (**A**) Microconidia of the wild-type strain (wt) were trapped in front of the channels at time 00:00. After 26 h 20 min, most wt hyphae had passed through the channels and continued to grow. Hyphal morphology in the channels or after passage in the *fmk1*Δ, *hog1*Δ, *mpk1*Δ, *gas1*Δ, *chs5*Δ, *tsc2*Δ, and *ck1*Δ mutants is shown. Enlarged images on the right show aberrant hyphal morphologies (marked with asterisks). Abnormal septation at narrow intervals is indicated by arrows. Scale bars, 20 µm. (**B**) Pass rate, defined as the percent of hyphae that passed through the channel and continued growing normally, was determined in the wt and the indicated isogenic gene deletion mutants. *n* > 100 hyphae in three independent experiments. Error bars (SD). *P < 0.05, **P < 0.01, vs wt according to Welch’s *t*-test. Only hyphae that entered the channel were counted for calculating pass rates. Mutants in components functioning in the Fmk1 IG, Mpk1 CWI, or Hog1 hyperosmolarity MAPK pathway are indicated in blue, orange, and green, respectively. (**C**) Schematic diagram of cellular components functioning in different morphogenetic processes of *F. oxysporum* such as IG, cell wall remodeling, asexual development, or virulence. Components tested in this study are shown in different colors. Elements of the three conserved MAPK pathways are shown in blue for the IG pathway, orange for the CWI pathway, and green for the Hog1 pathway. Elements with two colors are involved in multiple pathways. Arrows and bars indicate positive and negative interactions, respectively.

Previous work established that hyphae of *F. oxysporum*, as well as other filamentous fungi, can grow efficiently through a 1 µm wide channel, a space that is approximately a fourth of the average hyphal diameter (16). Here, we investigated the genetic bases of hyphal growth across narrow spaces, using a cross-shaped microfluidic device with a liquid medium flowing in a central inlet and exiting through four outlets with a total of 80 channels (Fig. S1A). The width of the channels, measured using a confocal microscope with a medium containing a fluorescent reagent, was 1.4 ± 0.2 µm, whereas the average width of the hypha was 4.3 ± 0.4 µm. (Fig. S1B and S1C). The height in the device was designed to be 4 µm, and the height of the space before and after the channel was also 4 µm (Fig. S1D), whereas the height within the channel was 2–3 µm. Aliquots of 50–100 *F. oxysporum* microconidia were inoculated at the center of the device, resulting in an average of 1–2 conidia trapped in front of each channel. After 6 h, hyphae invading the channels were captured at 20 min intervals through multipoint time-lapse imaging. As reported previously, hyphae of *F. oxysporum* crossed the channels with high efficiency (Fig. 1A). Approximately 90% of the wild-type hyphae passed the channels (*n* > 100 hyphae in three experiments), and occasionally multiple hyphae simultaneously traversed a single channel (Fig. 1A and B; Movie S1). After emerging from the channel, some wild-type hyphae slightly increased in width or temporarily stopped elongating, but most eventually continued elongating normally.

To examine the mechanisms underlying hyphal growth through an extremely narrow space, we tested a panel of isogenic *F. oxysporum* mutants affected in different morphogenetic processes such as IG, cell wall remodeling, asexual development, or virulence, for their ability to cross the channel (Fig. 1C). We first examined mutants lacking genes encoding components of the conserved IG MAPK cascade, which is essential for fungal pathogenicity in plants (10, 12). A mutant lacking the IG MAPK Fmk1 was able to pass the channel as efficiently as the wild-type strain (Fig. 1A and B; Movie S2). In line with this, single and double mutants in the mucin-like membrane protein Msb2 or the tetraspan transmembrane protein Sho1, two surface sensors functioning upstream of Fmk1 (7, 17), or a mutant lacking the homeodomain transcription factor Ste12 which regulates IG downstream of this MAPK (18) were also able to traverse the channel as efficiently as the wild type (Fig. 1A through C). We further noted that a mutant lacking the conserved MAPK Hog1, which mediates fungal responses to high osmolarity stress (19, 20), passed the narrow channel to a similar extent as the wild-type strain (Fig. 1A through C; Movie S3). Together, these results demonstrate that the conserved IG and hyperosmolarity response MAPK pathways are both dispensable for hyphal growth in confined spaces.

### The cell wall integrity MAPK cascade is required for efficient hyphal growth through microchannels

Fungi possess a third conserved MAPK signaling pathway, whose central component, the MAPK Mpk1, functions as a key regulator of the cell wall remodeling and integrity response (6, 20, 21). Here, we found that hyphal growth through the channels was largely blocked in a mutant lacking Mpk1, resulting in a significant (*P* < 0.01) reduction in the pass rate of around 70% (Fig. 1A through C; Movie S4). Furthermore, in some cases where *mpk1*Δ hyphae were able to traverse the channel, after exit from the narrow section, hyphal elongation was arrested, or aberrant swollen structures were formed (Fig. 1A, Fig. S1E).

Mpk1 functions as a conserved regulator of fungal CWI (21), and its growth is strongly impaired in the presence of the cell wall-targeting compound calcofluor white (CFW) or at high temperatures (Fig. S2). We, therefore, examined isogenic deletion mutants in genes known to function in cell wall biosynthesis and remodeling downstream of the Mpk1 pathway. Similar to *mpk1*Δ, mutants lacking the chitin synthases Chs5 or Chs7 (22, 23) or the β−1,3-glucanosyl transferase Gas1 (24) were impaired in hyphal elongation within the channel and exhibited significantly reduced pass rates (39%, 67%, or 30% of the wild type, respectively) as well as morphological alterations after the channel passage, including hyphal swellings or increased hyphal branching (Fig. 1A through C, Movies S5 and S6).

Besides its main role in the maintenance of CWI, the Mpk1 pathway cooperates with the Fmk1 cascade to control differentiation and developmental processes such as vegetative hyphal fusion (VHF). We, therefore, tested mutants lacking the Gal4-type transcription factor Pro1 or the signaling protein Fso1, both of which function downstream of Fmk1 and Mpk1 and are essential for VHF but dispensable for CWI (25, 26) (Fig. 1C). No significant differences with the wild-type strain were detected in the ability of *pro1*Δ or *fso1*Δ hyphae to cross the channels (Fig. 1B). Collectively, these results establish an essential role of the Mpk1 cascade during hyphal growth in confined spaces, which is closely linked to the function of this MAPK in CWI and remodeling.

### Additional signaling components contributing to hyphal growth through confined spaces

We next tested *F. oxysporum* mutants affected in a variety of signaling components involved in different aspects of hyphal growth, morphogenesis, development, and pathogenicity (Fig. 1C). The *fbp1* gene encodes an F-box protein that acts as a component of the E3 ubiquitin ligase in controlled protein degradation and contributes to the activation of the Mpk1 MAPK pathway in *F. oxysporum* (27). The *tsc2* gene encodes a GTPase-activating protein that represses activation of the global regulator of cell growth and development, Target Of Rapamycin Complex 1 (TORC1) (28). *F. oxysporum tsc*Δ mutants were previously shown to exhibit alterations in CWI (29). The *ck1* gene encodes a casein kinase that acts downstream of TORC1 and functions as a negative regulator of the essential plasma membrane H^+^-ATPase Pma1 (30). In *F. oxysporum*, Ck1 controls intracellular pH, activation of Mpk1, as well as hyphal growth and virulence (31). Here, we found that the hyphae of the *tsc2*Δ and *ck1*Δ mutants had reduced pass rates of 65% and 44% of the wild type, respectively, and often displayed abnormal morphologies within the channel (Fig. 1A and B; Movie S7). The fact that these mutants were previously reported to have defects in CWI and/or in Mpk1 regulation (27, 29, 31) further highlights the critical role of the CWI MAPK pathway in hyphal growth within microspaces. By contrast, mutants in *veA* or *velB* encoding two components of the conserved Velvet complex, which controls fungal development and secondary metabolism (32, 33), were unaffected in passage through the channel (Fig. 1B and C).

### Defects in cell wall remodeling cause the inability to reduce the hyphal diameter, thereby preventing growth into microspaces

**Fig 2:**
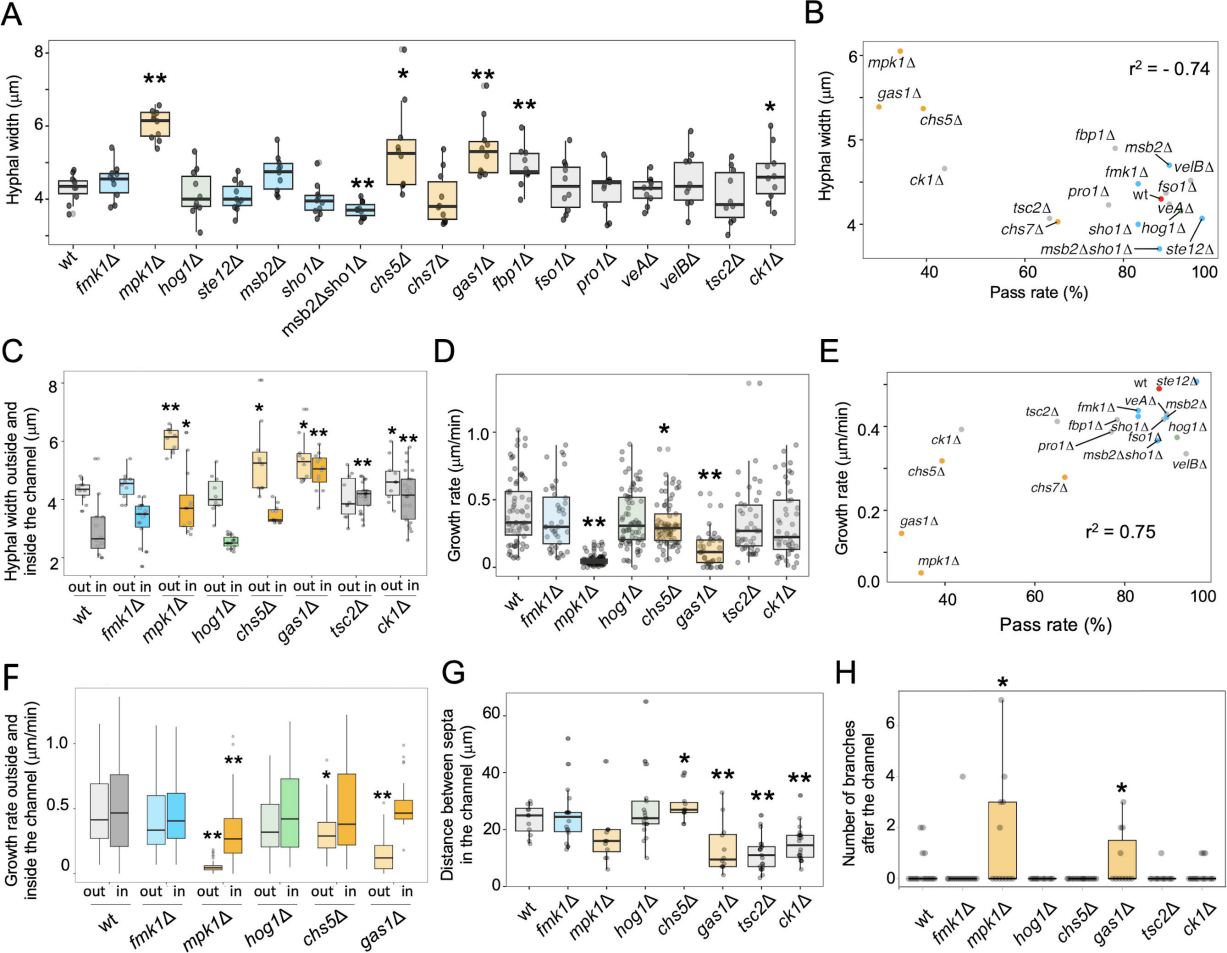
Hyphal width, growth rate, the distance between septa, and the number of branches of the different strains tested. (**A**) Boxplot showing hyphal widths of the indicated strains during growth outside the channel. *n* = 10. (**B**) Correlations between pass rates and hyphal width in the indicated strains. *r*^2^ = −0.74. (**C**) Boxplot showing hyphal widths outside or inside the channel. *n* = 10. (**D**) Boxplot showing hyphal growth rate outside the channel. *n* > 11. (**E**) Correlations between pass rates and growth rate of the indicated strains. *r*^2^ = 0.75. (**F**) Boxplot showing hyphal growth rate outside or inside the channel. *n* > 11. (**G**) Boxplot showing the distance between hyphal septa inside the channel. *n* > 11. (**H**) Boxplot showing the number of hyphal branches within 100 µm after passing the channel. *n* > 11. (**A–H**) *P < 0.05, **P < 0.01, vs wt according to Welch’s *t*-test. Mutants in components functioning in the Fmk1 IG, Mpk1 CWI, or Hog1 hyperosmolarity MAPK pathway are indicated in blue, orange, or green, respectively.

A comparison of the hyphal widths of the different strains revealed that the hyphae of the *mpk1*Δ, *chs5*Δ, *gas1*Δ, *fbp1*Δ, and *ck1*Δ mutants were significantly wider than those of the wild-type strain (Fig. 2A). Importantly, a negative correlation between hyphal width and channel passage rate was detected (Fig. 2B, S1F, *r*^2^ = −0.74). Furthermore, while the diameter of hyphae of the wild type and most other strains was reduced inside vs outside of the channel, the hyphal width of the *gas1*Δ, *tsc2*Δ, and *ck1*Δ mutants within the channel was significantly higher than those of the wild type (Fig. 2C), resulting in an expansion of the channel (Fig. 1A).

Hyphae of the *mpk1*Δ, *chs5*Δ, and *gas1*Δ mutants displayed significantly reduced elongation rates compared to those of the wild-type strain and other mutants (Fig. 2D, S1G). A positive correlation between hyphal elongation and channel passage rate was detected (Fig. 2E, S1H, *r*^2^ = 0.75). The hyphae of the wild type, *fmk1*Δ, and *hog1*Δ showed no significant difference in elongation rates inside or outside the channel, while the reduced hyphal elongation rates of *mpk1*Δ, *chs5*Δ, and *gas1*Δ mutants were partially restored within the channel (Fig. 2F). The number of septa in some of the hyphae increased within the channel, while the inter-septal distance was reduced in the *mpk1*Δ, *gas1*Δ, *fbp1*Δ, and *ck1*Δ mutants (Fig. 1A and 2G). After emergence from the channel, the *mpk1*Δ and *gas1*Δ mutants showed a significant increase in hyphal branching frequency (Fig. 1A and 2H). The other mutants did not show significant differences in the distance between septa or the number of branches.

### Cell wall chitin and glucan signal is reduced in the channels

**Fig 3:**
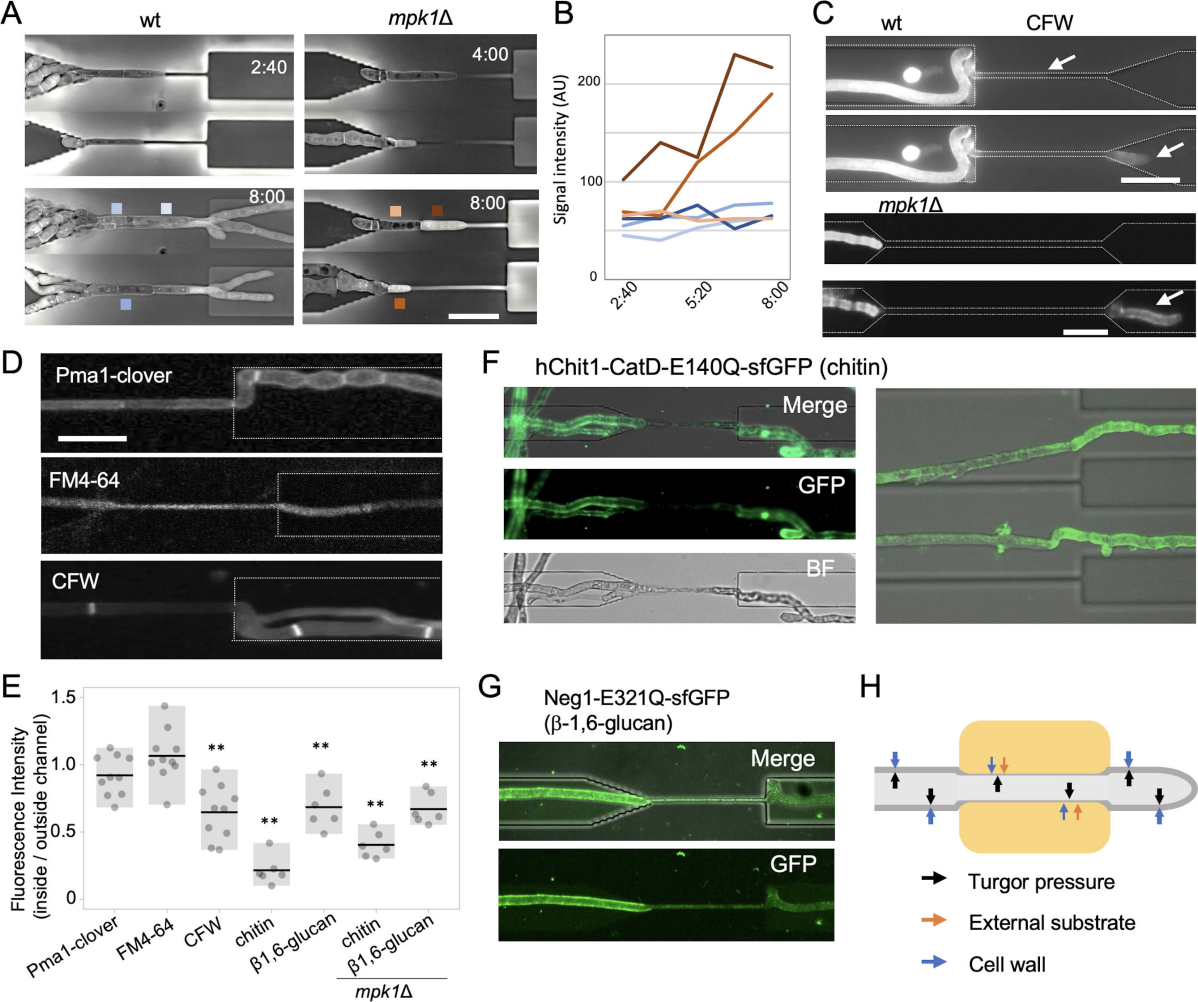
Cell wall alterations in the channel. (**A**) Time series by holographic microscopy of hyphae of the wt or the *mpk1*Δ strain growing inside the channel. The elapsed time indicated in h:min. Scale bars 20 µm. (**B**) Changes in signal intensity across time in the hyphae located close to the boxes shown in panel **A** colored either in blue (wt) or brown (*mpk1*Δ). (**C**) CFW staining of hyphae of the wt or the *mpk1*Δ mutant inside the channel (upper) or after passing the channel (lower). Hyphal tips are indicated by arrows. Scale bars 20 µm. (**D**) Fluorescent images showing hyphae growing inside or after exiting the channel, expressing PmaA-Clover or stained with FM4-64 or CFW. Scale bars, 20 µm. (**E**) The ratio of fluorescence intensity of hyphae inside vs outside the channel. *n* = 6–10, **P < 0.01, vs PmaA-Clover according to Welch’s *t*-test. (**F and G**) Fluorescence images of hyphae growing inside or after exiting the channel, grown in the presence of 5 µg/mL of the chitin- or β−1,6-glucan-binding domain fused with superfolder green fluorescent protein. (F, right) Fluorescence image of hyphae detached from the channel after removing the device from the slide glass. Scale bars, 20 µm. (**H**) Schematic diagram depicting structural changes in the cell wall of hyphae growing inside the channel.

We used holographic microscopy to capture the intrinsic refractive index of fungal cells, thus allowing label-free visualization of organelles and materials with higher refractive indexes in living cells (34). The hyphae of the wild-type strain showed no detectable changes in their refractive appearance after entering the channel and continued elongating normally, whereas those of the *mpk1*Δ mutant stopped elongating upon entering the channel, and the intracellular refractive index at the hyphal tip remained uniformly high with no distinct subcellular organelles visible (Fig. 3A and B, Movies S8 and S9). These results suggest that stress from invading confined spaces may induce significant structural changes in the tip apical cells and lead to arrest of hyphal elongation.

When hyphae of the wild type or the *mpk1*Δ mutant were stained with 5 µM of the chitin-binding dye CFW, the fluorescent signal was more intense on the outside than on the inside the channel (Fig. 3C and D). Furthermore, hyphae stained with chitin- or β1,6-glucan-specific binding protein-green fluorescent protein (GFP) markers (35, 36) exhibited a strongly reduced fluorescence signal inside compared to outside the channel, with the reduction of the chitin signal being more pronounced (Fig. 3E through G). By contrast, no differences in fluorescence signal intensity of the hyphae inside and outside the channel were detected using the membrane staining dye FM4-64 or a fungal strain expressing a fluorescently labeled version of the plasma membrane-localized H^+^-ATPase PmaA (31) (Fig. 3D and E). The former result suggests that the diffusion of the stains into the channel was not affected. Taken together, these results suggest a reduction in the cell wall chitin and glucan content of the hyphae growing inside the channel (Fig. 3H).

The hyphae of the *mpk1*Δ mutant also showed a reduction of chitin- and glucan-specific signals within the channel, as well as a more uneven distribution of the fluorescence along the hyphae outside the channel compared to the wild type (Fig. 3E, Fig. S1I). Detachment of the device from the bottom glass revealed that those hyphae that had exited the channel also had a lower fluorescence signal (Fig. 3F, right).

### High extracellular osmolarity restores colony growth and channel pass rate in cell wall integrity mutants

**Fig 4:**
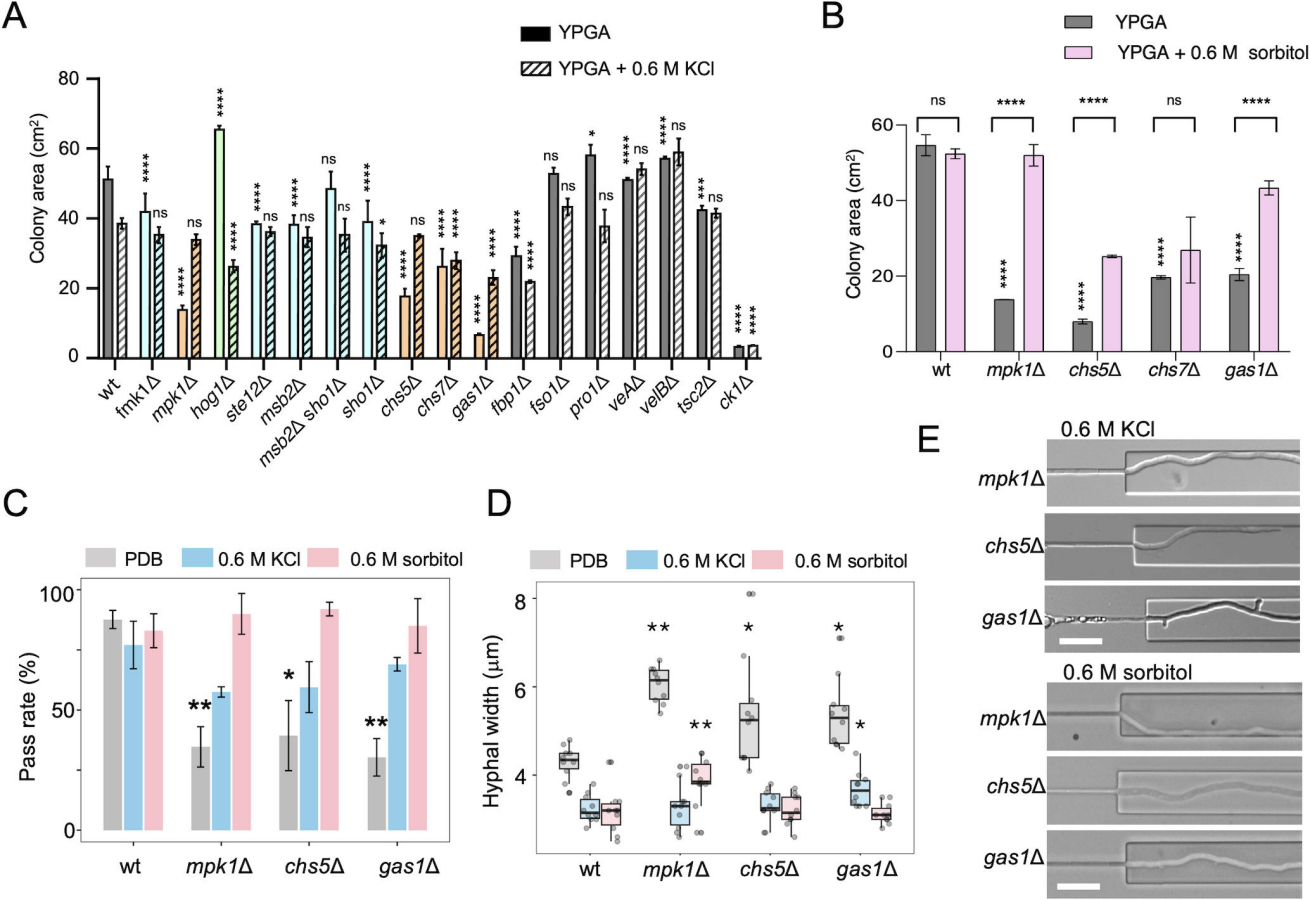
High extracellular osmolarity restores colony growth and channel pass rate in CWI mutants. (**A and B**) Aliquots of 5 × 10^4^ fresh microconidia of the indicated strains were spot inoculated on YPGA medium either lacking or supplemented with 0.6 M KCl (**A**) or 0.6M sorbitol (**B**). The colony area after 7 days at 28°C was measured using the Image J software. *P < 0.05, **P < 0.01, ****P < 0.0001 vs wt according to Welch’s *t*-test. The data shown are the mean from three independent plates per strain. (**C**) Pass rates (%) of the wt, *mpk1*Δ, *gas1*Δ, or *chs5*Δ grown in potato dextrose broth (PDB; gray), or in PDB supplemented with 0.6 M KCl (blue) or 0.6 M sorbitol (pink). *n* > 100 hyphae in three independent experiments. *P < 0.05, **P < 0.01, vs wt according to Welch’s *t*-test. (**D**) Boxplot showing hyphal widths in the wt, *mpk1*Δ, *gas1*Δ, or *chs5*Δ grown either in PDB (gray) or in PDB supplemented with 0.6 M KCl (blue) or 0.6 M sorbitol (pink). *n* = 10. (**E**) Hyphal morphology of the *mpk1*Δ, *gas1*Δ, or *chs5*Δ mutants after exit from the channels under high osmolarity conditions with 0.6 M KCl or sorbitol. Scale bar, 20 µm.

Previous work established that morphogenetic defects in fungal CWI mutants can be rescued by increasing extracellular osmolarity and thereby reducing turgor pressure on the compromised cell wall (37). Here, we found that the reduced colony growth of the *mpk1*Δ, *gas1*Δ, *chs5*Δ, and *chs7*Δ mutants on YPGA plates could be restored to wild-type levels by adding 0.6 M KCl or sorbitol to increase external osmolarity (Fig. 4A and B, Fig. S3). Importantly, an increase in extracellular osmolarity, 0.6 M KCl or sorbitol, also rescued the channel pass rates of these CWI mutants to levels similar to those of the wild type (Fig. 4B and C). Concomitantly, hyphal width decreased to values resembling those of the type strain (Fig. 4D and E). Taken together, these results suggest that reduction of the hyphal turgor pressure on the compromised cell wall by increasing extracellular osmolarity restores the ability of the CWI mutants to narrow their hyphal diameter, thereby facilitating their passage through the channels.

### Cell wall-dependent entry into microspaces and invasive hyphal growth across cellophane membranes independently contribute to fungal pathogenicity

**Fig 5:**
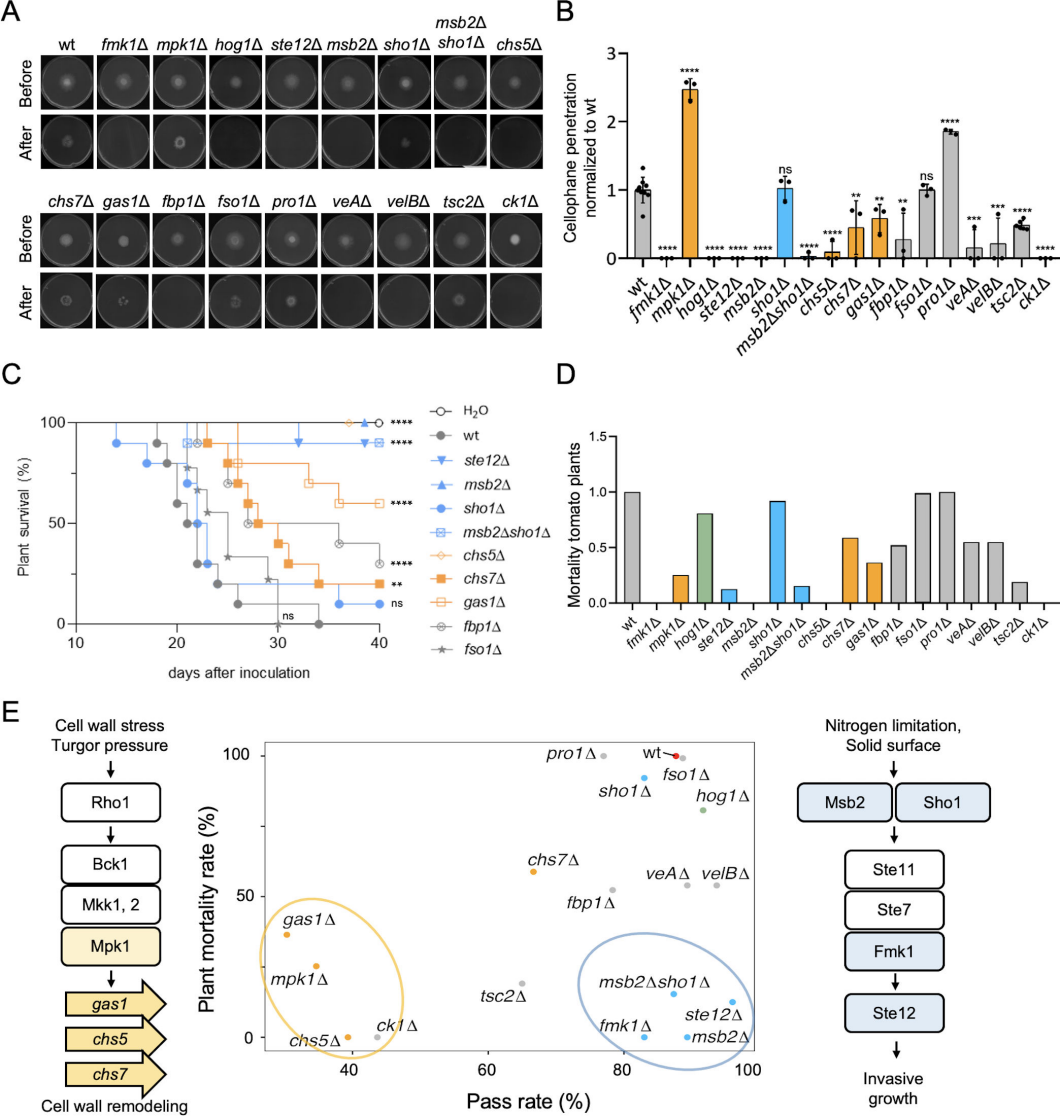
Cell wall remodeling and infection-related morphogenesis contribute independently to fungal pathogenicity in plants. (**A**) IG is measured by penetration across cellophane membranes. The indicated strains were spot inoculated on a plate with minimal media (MM) covered with a cellophane membrane (before). After 3 days plates were imaged (before), then the cellophane with the fungal colony was removed, and the plates were incubated for two additional days to visualize the presence of mycelium that had penetrated through the cellophane (after). The images shown are representative of three independent plates for each strain. (**B**) Quantification of cellophane penetration in the indicated mutants, normalized to wild type. Mutants in components of the three conserved MAPK pathways are shown in blue for the IG pathway, orange for the CWI pathway, and green for the Hog1 pathway. (**C**) Kaplan-Meier plots showing the survival of groups of 10 tomato plants (cv. Moneymaker) whose roots were dip-inoculated with a suspension of 5 × 10^6^ microconidia/mL of the indicated strains. Percentage survival was plotted for 40 days. ****P < 0.0001, **P < 0.01 vs wt according to log-rank test. ns, non-significant. (**D**) The area over the curve calculated from Kaplan-Meier plots as a measure of mortality in tomato plants caused by the indicated mutants, normalized to wild type. (**E**) The correlation between mortality caused on tomato plants (see D) and pass rate through the channels (Fig. 1B) was calculated for each strain. Components of the Mpk1 CWI and the Fmk1 IG MAPK pathways shown in the diagrams on the left and the right are circled in orange and blue, respectively.

Previous work established a key role of invasive hyphal growth, quantified as the ability to cross a cellophane membrane, in plant infection (13). Here, we found that the *fmk1*Δ, *msb2*Δ, *msb2*Δ*sho1*Δ, and *ste12*Δ mutants, which lack key components of the IG MAPK cascade, were unaffected in channel pass rate (Fig. 1A and B) but severely impaired in cellophane penetration (Fig. 5A and B).

We next examined the correlation between the abilities of the different strains to pass the channels and to cause mortality on the host plant tomato. The *fmk1*Δ, *ste12*Δ, *msb2*Δ, and *sho1*Δ mutants, which were unaffected in channel pass rate, caused significantly less mortality in tomato plants than the wild-type strain (Fig. 5C through E) (20). On the other hand, the *mpk1*Δ, *gas1*Δ, and *tsc2*Δ mutants, which were severely impaired in channel pass rate but unaffected in cellophane penetration, also caused reduced mortality in tomato plants (Fig. 5C through E) (20, 29). Finally, the *chs5*Δ and *ck1*Δ mutants, which were impaired both in channel pass rate and in cellophane penetration, were essentially non-pathogenic (Fig. 5C through E) (31). Taken together, these results suggest that cell wall remodeling is crucial both for the capacity of hyphae to invade microspaces as well as for plant infection, while invasive hyphal growth has an independent role in pathogenicity that appears to be unrelated to the capacity to grow within narrow spaces.

Root infection experiments were conducted to compare the growth of the hyphae of the wild type and of CWI mutants in liquid culture and during the invasion of tomato roots. Wheat germ agglutinin (WGA)-Alexa Fluor staining of the fungal cell walls revealed abnormal hyphal growth and reduced cell wall chitin content in the mutants *mpk1*Δ and *gas1*Δ as well as aberrant morphologies with balloon-like structures in the *chs5*Δ mutant as previously reported (22) and a complete lack of root colonization in the *chs7*Δ mutant (Fig. S4).

## DISCUSSION

In the present study, we systematically analyzed a panel of isogenic mutants of the fungal pathogen *F. oxysporum* for their capacity to invade and grow through microchannels. While the wild-type strain was able to successfully pass the channel and resume growth after channel exit, several mutants were strongly impaired in different steps of this process. One critical factor was the capacity to adjust the hyphal diameter to the width of the channel, which is much narrower than the average width of a fungal hypha. In line with a previous study (16), successful elongation across the channel was associated with a marked decrease in hyphal diameter, confirming the high morphogenetic plasticity of *F. oxysporum* hyphae. Several mutants compromised in CWI displayed increased hyphal diameter under physiological conditions and were severely affected in pass rate across the channels, strongly suggesting a functional link between these two parameters. Further supporting this idea, an increase in external osmolarity restored both the capacity of these mutants to reduce hyphal width and to pass through the narrow channel. We further noted a visible widening of the microchannels as the wild-type hyphae extended through the channel space, suggesting the presence of a high turgor pressure within these filamentous structures. Young’s modulus of polydimethylsiloxane (PDMS) used for channel fabrication is comparable to that of the cell wall of maize root vascular parenchyma (38, 39). However, Young’s modulus of *F. oxysporum* hyphae is higher than that of PDMS or plant cell walls (16), suggesting that a similar mechanism of turgor-mediated displacement of plant cell walls could apply during the growth of *F. oxysporum* hyphae in the apoplast of the root cortex. In support of this, fluorescence microscopy analysis of a GFP-marked *F. oxysporum* strain revealed displacement of root cell walls by the fungal hyphae extending into the intercellular spaces of plant cells (40). While these results provide preliminary evidence for the role of cell wall remodeling and turgor pressure during infection, live cell imaging of hyphal extension between root cells remains challenging. Using a microfluidic device, we established that the reduced channel pass rates of CWI mutants are restored by a rise in external osmolarity. This highlights the importance of cell wall remodeling and internal turgor pressure during hyphal growth into microspaces and provides a possible explanation for the importance of the CWI MAPK cascade in fungal infection of plants (41).

Conversely, the IG MAPK cascade, which is crucial for plant infection, was dispensable for growth into microspaces since isogenic mutants lacking key components of this pathway exhibited channel pass rates similar to those of the wild-type strain. This finding was somewhat unexpected, given that the conserved Fmk1 MAPK cascade is essential for the successful penetration of cellophane membranes by *F. oxysporum* (13). Moreover, the homologous MAPK Pmk1 of the rice blast fungus *Magnaporthe oryzae* is required for *in planta* cell-to-cell movement across pit fields, which are composed of extremely narrow plasmodesmata (14). However, in line with our findings here, Pmk1 inactivation did not affect the morphology of *M. oryzae* invasive hyphae, which still formed terminal swellings at host wall contact points. In stark contrast to the present study, selective inactivation of the CWI MAPK Mps1 by a chemical inhibitor did not block the cell-to-cell movement of *M. oryzae* across plasmodesmata, suggesting that cell wall remodeling is dispensable for this process (14). This apparent contradiction could be explained by structural differences between the microfluidic channels used in the present study and the rice plasmodesmata or by only a partial effect of the chemical MAPK inhibitor used in the study on the *in planta* activity of Mps1.

It has been suggested that plant cells trigger an immune response upon hyphal invasion, and plant-infecting fungi respond to such external insults from host defense mechanisms (42, 43). While the microfluidic device used in the present study mimics the physical aspects of hyphal growth in a confined space, it lacks the chemical effects associated with plant defense responses. Interestingly, the chitin and glucan content in the cell wall decreased markedly during hyphal growth in the microchannel. This might reflect the role of cell wall remodeling in balancing the internal hyphal turgor pressure against the external force exerted by the channel to preserve the structural integrity of the fungal cell wall (Fig. 3G). However, it is possible that hyphal growth, branching, and morphology within plant tissues may involve additional, more complex cell wall modifications. This highlights the need for further analysis of structural changes of fungal hyphae in the plant environment.

Our genetic analysis suggests that the Mpk1 and the Fmk1 MAPK cascades have distinct, yet complementary roles during infection. While the former mediates cell wall remodeling, which is required for hyphal growth in the restricted space between plant cells, the latter is linked to hyphal direction and differentiation during the infection process. Thus, while both pathways play essential roles in pathogenicity, each pathway independently contributes to the infection process. Considering that hyphal growth between living plant cells is not only crucial for phytopathogens but also for symbionts such as plant arbuscular mycorrhizal fungi (44), the use of microfluidic devices could prove highly useful for analyzing hyphal growth in such challenging biological systems (45). We recently succeeded in creating even narrower microchannels with a width of 0.5 µm and pores as small as 0.3 µm. Future efforts will be directed toward analyzing the impact of decreasing channel width or increasing the stiffness of PDMS on hyphal passage rates and cell wall modifications. Because microfluidic devices offer flexibility in design, microspaces resembling real plant cells could be created in a brick-like configuration, making it feasible to quantify mycelial entry and extension into microspaces, as well as other developmental processes such as transpressorium formation, hyphal branching, or tip growth (46). We recently developed a system to measure hyphal chemotropism using a newly designed microfluidic device (47). Microfluidic devices thus have the potential to improve our knowledge of the conserved principles governing hyphal growth and lead to a better understanding of the role of these filamentous structures in fungal pathology, ecology, and biotechnology.

## MATERIALS AND METHODS

### Fungal strains and culture conditions

The *F. oxysporum* mutants used in this study are listed in Table S1. All are derived from the wild-type isolate *F. oxysporum* f. sp. *lycopersici* 4287 (NRRL34936). Strains were grown in potato dextrose broth (PDB) supplemented with the appropriate antibiotic(s) at 28°C and 170 rpm as described (48) and stored as microconidial suspensions at −80°C in 30% (vol/vol) glycerol.

### Microfluidic device

The microfluidic devices originally designed for culturing tip-growing plant cells (49) were adapted for the current fungal cell studies as described (16). Briefly, photoresist (SU-8 3005 and 3010) based microstructures were created on a silicon wafer using a maskless lithography system (DL-1000; Nano System Solutions, Inc.). The PDMS precursor (Silicone Elastomer Base, SYLGARD) and curing agent (Silicone Elastomer Curing Agent, SYLGARD) in a 10:1 ratio in a plastic cup and mixed vigorously with a medicine spoon for about 5 min to allow air to enter the mixture. After curing at 60°C for 24 h, PDMS was cut from the substrate using a cutter, and holes were drilled in the culture medium inlet and outlet portions at a total of five locations using a 2 mm diameter biopsy trepan (KAI industry). PDMS was bonded to the cover glass by plasma treatment (CUTE, Femoto Science).

### Microscopy

Cells were observed using an epifluorescence inverted Axio Observer Z1 (Carl Zeiss) microscope equipped with a Plan-Apochromat 20× lens objective, an AxioCam 506 monochrome camera, and Colibri.2 LED lights (Carl Zeiss). A confocal laser scanning microscope LSM880 (Carl Zeiss) equipped with a 63×/0.9 numerical aperture Plan-Apochromat objective and a 40×/0.75 numerical aperture IR Achroplan W water immersion objective (Carl Zeiss) was used to acquire confocal microscopic images. Images were collected and analyzed by the Zen system (Carl Zeiss) and ImageJ software.

### Microfluidic device imaging

A pre-culture was done in a small test tube with 5 mL PDB medium inoculated from a microconidia suspension stock stored at 30°C and incubated at 180 rpm for 2–3 days with shaking. Antibiotics were added as required (*fmk1*Δ, Phleomycin 4 µg/mL; remaining mutants, Hygromycin B 20 µg/mL). The precultured medium was passed through a 20 µm filter (As One, Cell Fractionation Filter 20 µm, Filcon N Syringe 2-7208-01) to remove hyphae. The microconidia suspension was concentrated by centrifugation, diluted, and pipetted into the inlet at the center of the device. A total of 5 mL of PDB medium was placed into a 10 mL plastic syringe (SS-10ESZ, Terumo) and connected to a polyethylene tube (inner diameter 0.38  mm, outer diameter 1.09  mm; BD intramedic). After pushing the air from the syringe, the tube was infused into the inlet of the PDMS device. The number of microconidia flowing to the front of the microfluidic channel was adjusted by observing under the microscope. PDB medium was pumped from the syringe through the tubing into the device using a positive displacement syringe pump (YSP-101, YMC) at a rate of 12 mL/h. The device was fixed and incubated on a thermo-plate set at 25°C (TOKAI HIT). By using the inverted microscopy Axio Observer Z1 with a 20× objective lens, 10 channels fit in one view. Eight positions were set per device for the motorized stage, and three *z*-stack positions (interval 3 µm) were set for each position. Microconidia started germinating 6 h after inoculation. Hyphal growth in each channel was imaged every 20 min for 24 h by multipoint *z*-stack time-lapse. The solution coming out of the outlet was sucked up with a tissue. Images were collected and analyzed by the Zen system (Carl Zeiss) and ImageJ software. The diameter of the hyphae was measured by ZEN. The growth speed of the hyphae was measured with MtrackJ, a plug-in for ImageJ. The coordinates of the hyphal tip and its distance were recorded every 40 min to obtain the elongation rate of hyphae.

### Polysaccharide-binding proteins

Recombinant proteins for labeling polysaccharides exposed on the cell wall were prepared as well as previously reported with slight modifications (35, 36). Briefly, the recombinant endo-β−1,6-glucanase mutant (Neg1-E321Q) and the modified catalytic domain (CatD) of human chitotriosidase (hChit1-CatD-E140Q) were used as proteins that bind to the β−1,6-glucan and chitin structures, respectively. For fluorescent staining, a superfolder green fluorescent protein was fused to the C-terminus of each modified enzyme across the linker peptide (Gly-Gly-Ser-Gly-Gly-Gly-Ser-Gly-Gly) sequence. The purified protein stock was 200 µg/mL, and staining was performed at a final concentration of 5 µg/mL.

### Holographic microscopy

The same microfluidic device samples of wild type and *mpk1*Δ were observed using inverted holotomography microscopy in an HT - X1 (Tomocube) equipped with a 40 × 0.95 air lens objective and a 450 nm LED light. Samples were incubated at 30°C and imaged every 80 min for 24 h by multipoint time-lapse. PDB medium was not infused into the PDMS devices. Images were collected and analyzed by TomoStudioX software (Tomocube).

### Colony growth assays

To measure fungal colony growth, aliquots of 5 × 10^4^ fresh microconidia were spot inoculated onto YPGA plates (1% yeast extract, 2% tryptone, 2% glucose, and 2% agar), with or without supplementation with the osmotic stabilizers KCl or sorbitol (0.6 M). After the indicated days of growth at 28°C, plates were imaged, and the colony area was quantified using the Multigauge V3.0 software. The polygalacturonase production plate assay was carried out as described previously on polygalacturonic acid (PGA) medium (0.5% [wt/vol] PGA, 2% glucose, 0.2% ammonium sulfate, and 1.5% agar) adjusted to pH 7 by spot inoculating 10^5^ fresh microconidia (18). After 3 days at 28°C, the PGA was precipitated by adding 0.4 M HCl, the plate was imaged, and the area of the contrasting halo underneath the fungal colony was quantified using the Multigauge V3.0 software. For stress tolerance analyses, serial dilutions of fresh microconidia were spot inoculated on YPGA plates in the absence or presence of the following concentrations of stress-inducing compounds: SDS (12.5 mg/mL), CFW (50 µg/mL), KCl (1.2 M), and sorbitol (0.6 M; all from Sigma-Aldrich). Plates were incubated for 2 days at 28°C and imaged. Experiments were performed at least two times, each with three replicate plates.

### Invasive growth and plant infection assays

Invasive growth assays on cellophane membranes were performed as described (13). Briefly, MM plates were covered with a cellophane membrane, and 5 × 10^4^ fresh microconidia were spot-inoculated on top. After 3 days at 28°C, the cellophane membrane with the fungal colony was carefully removed, and plates were incubated for 2 additional days. Plates were imaged before and after cellophane removal. For the calculation of the invasive growth index, the area of fungal growth after removing the cellophane was measured using the Multi-Gauge V 3.0 software and normalized to the colony area before cellophane removal, and data were normalized to the wild-type strain. Experiments were performed at least two times, each with three replicate plates.

Tomato root inoculation assays were performed in a plant growth chamber (14/10 h, light/dark cycle, 28°C) as described (48) using the susceptible cultivar Moneymaker. Briefly, 2-week-old tomato plants were inoculated with the *F. oxysporum* strains by immersing the roots for 30 min in a suspension of 5 × 10^6^ microconidia/mL, planted in minipots with vermiculite and maintained in a plant growth chamber. Ten plants were used per treatment. Plant survival was recorded daily for 40 days (13). Survival was calculated by the Kaplan-Meier method and compared among groups using the log-rank test. Mortality was quantified as follows: area under the curve (AUC) of water treatment (negative control) minus AUC of the strain of interest. Values were normalized to that of the wild-type strain. Mortality data for the *fmk1*Δ, *mpk1*Δ, *hog1*Δ, *pro1*Δ, *veA*Δ, *velB*Δ, *tsc2*Δ, and *ck1*Δ mutants were taken from published experiments conducted under the same conditions described above (19, 20, 25, 31, 33).

For fluorescence microscopy analysis of root infection, the roots of tomato seedlings were dip inoculated with microconidia of *F. oxysporum* strains as indicated above and placed on water agar plates for 2 days. Fungal cell walls were stained with WGA-Alexa Fluor 488, and plant cell walls were stained with propidium iodide as described (50). Wide-field fluorescence imaging was performed with a Zeiss Axio Imager M2 microscope equipped with a Photometrics Evolve EMCCD camera, using the 40× oil objective.

## SUPPLEMENTAL MATERIAL

Supplemental material mbio.01184-25-s0001.pdf Fig. S1-4, Tables S1 and S2, and movie legends.

Movie S1 mbio.01184-25-s0002.avi *F. oxsporum* wt strain hyphae grown in the channels.

Movie S2 mbio.01184-25-s0003.avi *F. oxsporum fmk1*Δ strain hyphae grown in the channels.

Movie S3 mbio.01184-25-s0004.avi *F. oxsporum hog1*Δ strain hyphae grown in the channels.

Movie S4 mbio.01184-25-s0005.avi *F. oxsporum mpk1*Δ strain hyphae grown in the channels

Movie S5 mbio.01184-25-s0006.avi *F. oxsporum chs5*Δ strain hyphae grown in the channels.

Movie S6 mbio.01184-25-s0007.avi *F. oxsporum gas1*Δ strain hyphae grown in the channels.

Movie S7 mbio.01184-25-s0008.avi *F. oxsporum tsc2*Δ strain and *ck1*Δ strain hyphae grown in the channels.

Movie S8 mbio.01184-25-s0009.avi *F. oxsporum* wt strain hyphae grown in the channels imaged by holographic microscopy and DIC.

Movie S9 mbio.01184-25-s0010.avi *F. oxsporum mpk1*Δ strain hyphae grown in the channels imaged by holographic microscopy and DIC.

ASM does not own the copyrights to Supplemental Material that may be linked to, or accessed through, an article. The authors have granted ASM a non-exclusive, world-wide license to publish the Supplemental Material files. Please contact the corresponding author directly for reuse.
