# Supplementary material for: Cell wall remodeling in a fungal pathogen is required for hyphal growth into microspaces
Source: mBio. 2025 Jun 30;16(8):e01184-25. doi: 10.1128/mbio.01184-25 (PMC12345142; doi:10.1128/mbio.01184-25)
Supplement: Supplemental material — Fig. S1-4, Tables S1 and S2, and movie legends. [file mbio.01184-25-s0001.pdf]

Fig. S1

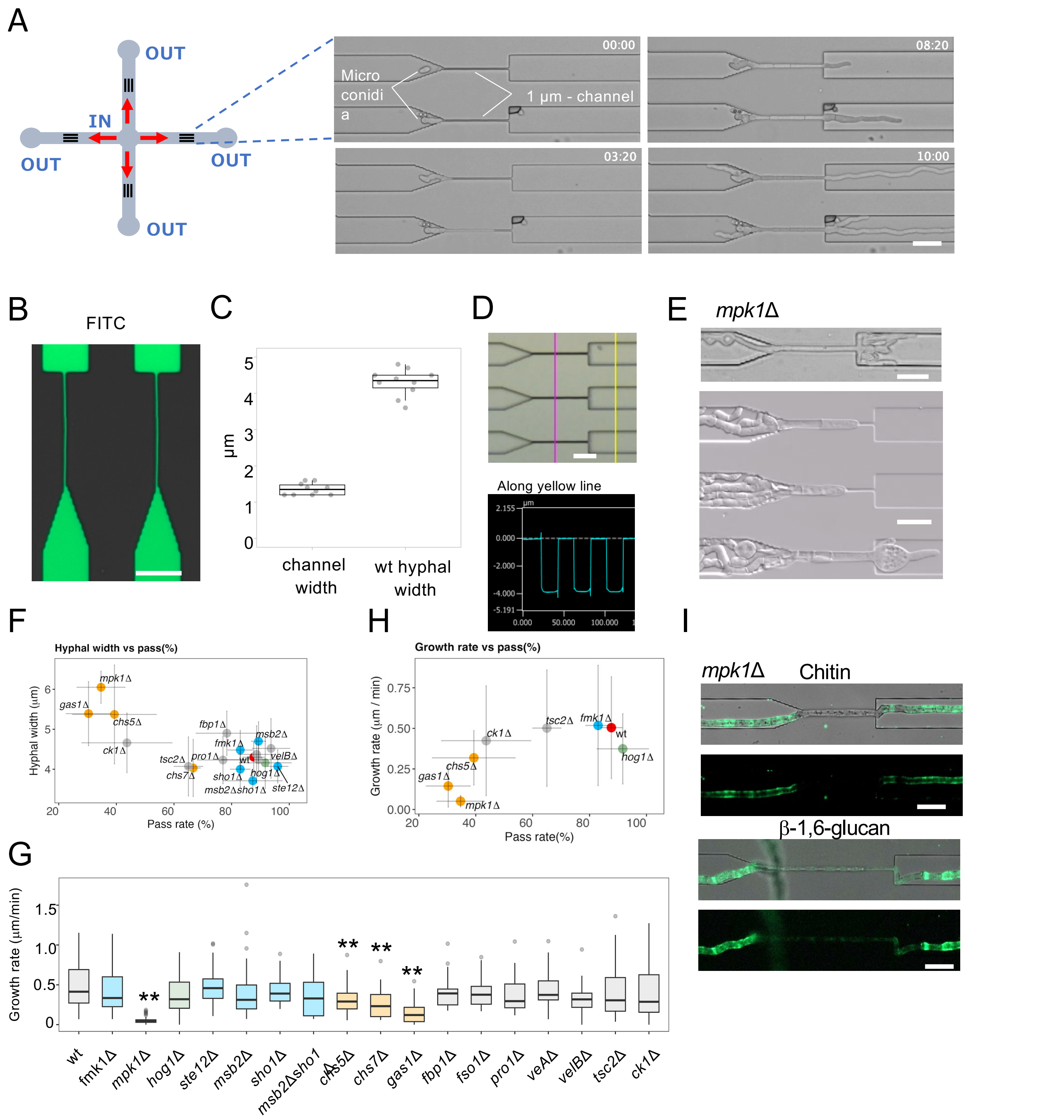

Figure S1. (A) Design of the microfluidic device with an inflow at the center (IN) and outflows at the four ends (OUT). Each arm of the cross-shaped design includes twenty microchannels, 1 µm in width and 50 µm in length, positioned between the inlet and outlet. Time series showing a hypha of *F. oxysporum* wt growing into the channel. The elapsed time is given in hours : minutes. (B) Confocal image of a solution containing FITC flowed into the microchannel. (C) Box plot of channel width and wt hyphal width measured by confocal images. n = 10. (D) Ultra-deep surface profile measurement using the laser microscope VK-X100 (Keyence). (E) Hyphal images in the channels or after passage in the *mpk1*Δ strain are shown. (F) Correlations between pass rates and hyphal width in the indicated strains. Error bars indicate SD. (G) Boxplot showing hyphal widths of the indicated strains during growth outside the channels. ** $p < 0.01$, versus wt according to Welch's t-test. (H) Correlations between pass rates and growth rate in the indicated strains. Error bars indicate SD. (I) Fluorescent images of hyphae of the mpk1Δ strain inside the channel and after passing the channel grown with chitin or β-1,6-glucan binding protein fused with sfGFP at 5 µg/ml. (A, B,D, E, I) Scale bars 20 µm.

Fig. S2

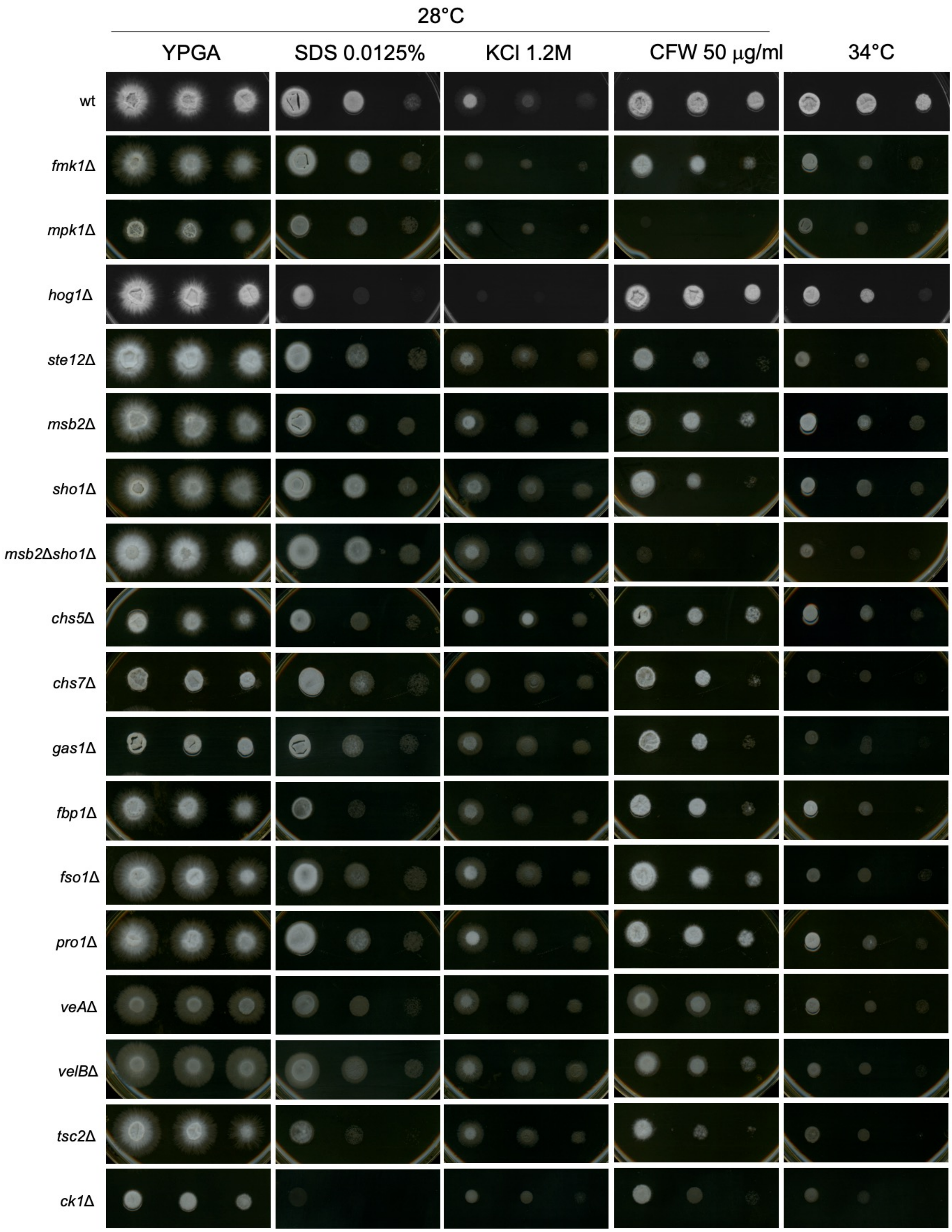

Figure S2. Colony growth phenotypes of the *Fusarium oxysporum* wild type strain and the indicated mutants on YPGA, either lacking or supplemented with the indicated stress compounds**.** Serial dilutions of fresh microconidia were spot-inoculated, plates were incubated at 28ºC in the dark and scanned after three days. Images shown are representative of three independent plates for each strain and growth condition.

Fig. S3

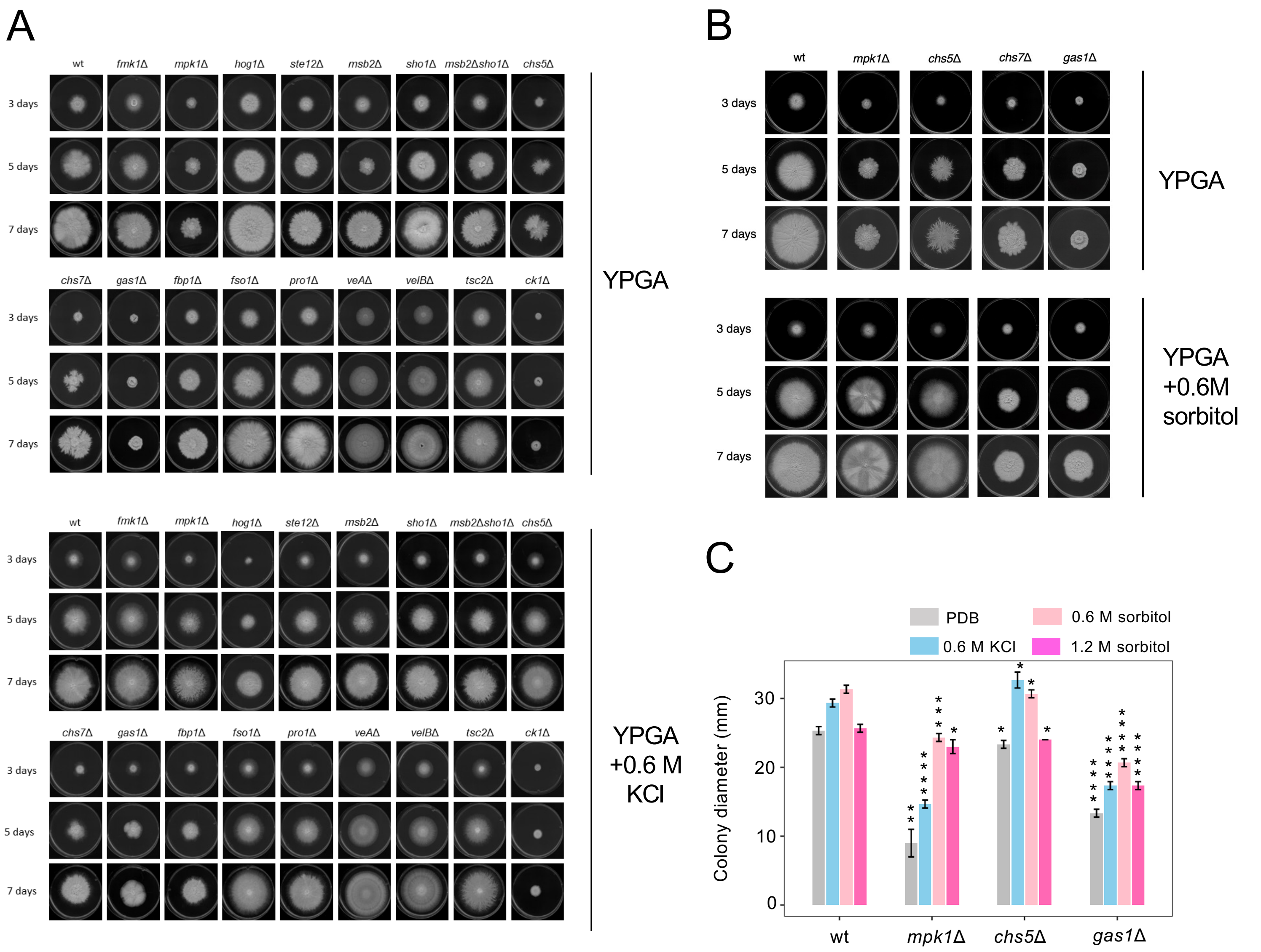

Figure S3. (A, B) Colony growth phenotypes of the *Fusarium oxysporum* wild type strain and the indicated mutants on YPGA, either lacking or supplemented with the osmotic stabilizer 0.6 M KCl or 0.6 M sorbitol. Aliquots of 5x10[4] fresh microconidia were spot-inoculated at the center, plates were incubated at 28ºC in the dark and scanned after 3, 5 and 7 days. Images shown are representative of three independent plates for each strain and growth condition. Note that from day 5 on, the colony growth of the *chs5*Δ and *chs7*Δ mutants on YPGA is masked by the appearance of fast-growing variant sectors. (C) (B) Colony diameter of wt, *mpk1*Δ, *gas1*Δ, and *chs5*Δ strains on PDB, + 0.6M KCl, + 0.6M sorbitol or + 1.2M sorbitol after 3 days. n = 3, error bars (SD). * $p < 0.05$, ** $p < 0.01$, **** $p < 0.0001$ versus wt according to Welch's t-test. Data shown are the mean from three independent plates per strain.

Fig. S4

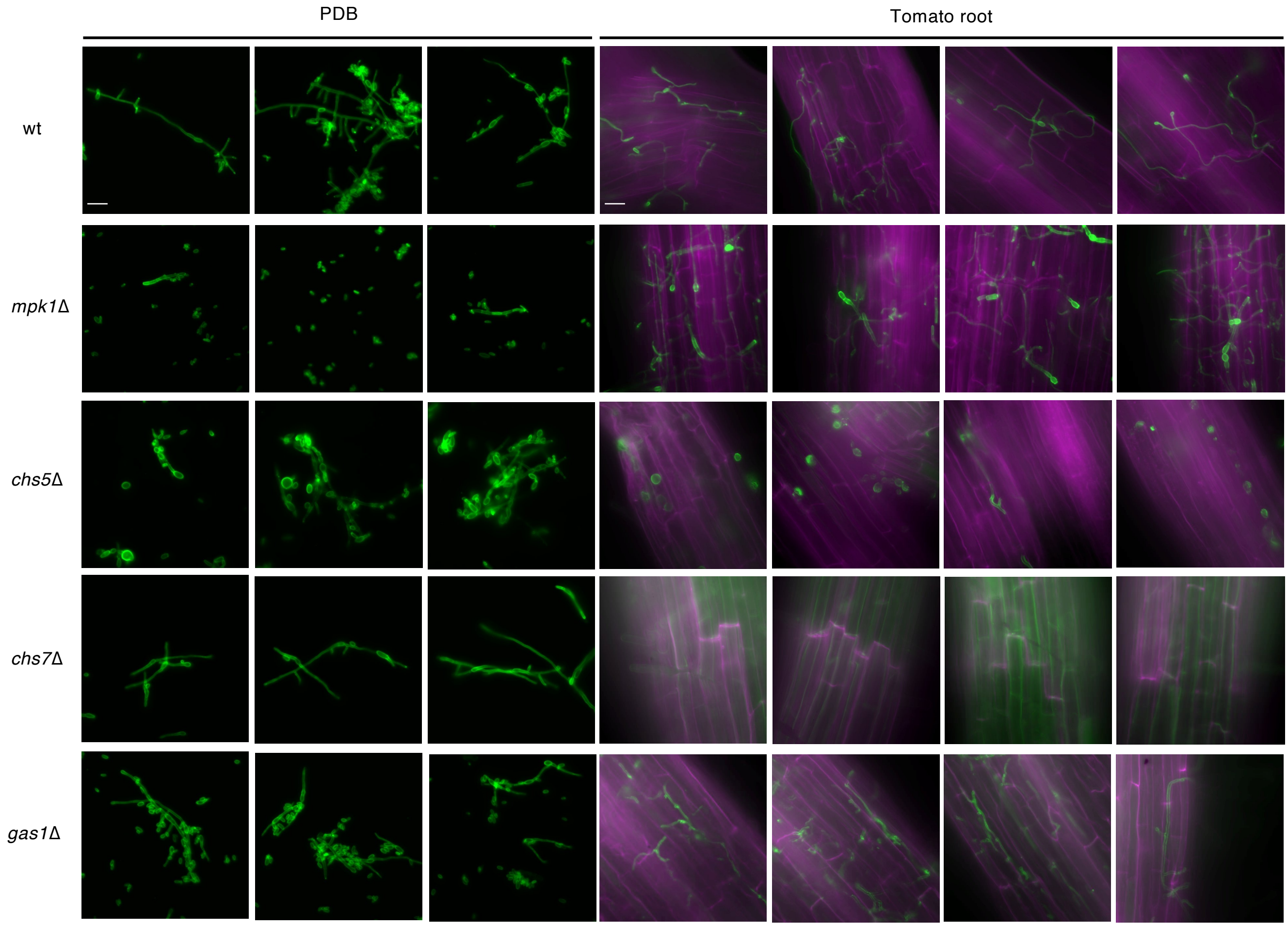

Figure S4. Fluorescence microscopy of the indicated *F. oxysporum* isolates during growth in liquid potato dextrose broth medium or during colonization of tomato roots at 2 days after inoculation. Fungal cell walls were stained with WGA-Alexa Fluor 488 (green) while plant cell walls were stained with propidium iodide (violet). Scale bars, 25 μm.

Table S1. *Fusarium oxysporum* strains used in this study.

Strain	*Predicted function*	*Phenotype complemented**	*Reference*
*ste12*Δ	Homeodomain transcription factor	Yes	(Rispail & Di Pietro, 2009)
*fmk1*Δ	Invasive growth mitogen-activated protein kinase	Yes	(Di Pietro *et al.*, 2001)
*mpk1*Δ	Cell wall integrity mitogen-activated protein kinase	Yes	(Turrà *et al.*, 2015)
*hog1*Δ	Hyperosmolarity mitogen-activated protein kinase	Yes	(Segorbe *et al.*, 2017)
*ck1*Δ	Casein kinase 1	Yes	(Mariscal *et al.*, 2022)
*pro1*Δ	Gal4-type transcription factor	Yes	(Palos-Fernández *et al.*, 2022)
*tsc2*Δ	Tuberous Sclerosis Complex 2, negative regulator of Target of rapamycin complex 1	Yes	(Navarro-Velasco *et al.*, 2023)
*gas1*Δ	β-1,3-Glucanosyltransferase	No	(Caracuel *et al.*, 2005)
*fso1*Δ	Fusarium so1, regulator of hyphal fusion	Yes	(Prados Rosales & Di Pietro, 2008)
*fbp1*Δ	F-box protein	Yes	(Miguel-Rojas & Hera, 2013)
*chs7*Δ	Chaperone-like protein	No	(Martín-Udíroz *et al.*, 2004)
*chsV*Δ	Class V chitin synthase	Yes	(Madrid *et al.*, 2003)
*msb2*Δ	Mucin	Yes	(Pérez-Nadales & Di Pietro, 2011)
*veA*Δ	Component of the Velvet regulatory complex	Yes	(Lopez-Berges *et al.*, 2013)
*velB*Δ	Component of the Velvet regulatory complex	Yes	(Lopez-Berges *et al.*, 2013)
*sho1*Δ	Transmembrane protein	Yes	(Perez-Nadales & Di Pietro, 2015)
*msb2*Δ/ *sho1*Δ	Double mutant	No	(Perez-Nadales & Di Pietro, 2015)

*Complementation of mutant phenotype by re-introduction of the wild type allele was confirmed in the original publication cited.

Table S2. Summary of phenotypes.

strain	pass rate	cellophane penetration	mortality
wt	+++	++	+++
fmk1Δ	+++	-	-
ste12Δ	+++	-	-
msb2Δ	+++	-	-
sho1Δ	+++	++	+++
msb2Δsho1Δ	+++	-	-
mpk1Δ	-	+++	+
chs5Δ	-	-	-
chs7Δ	+	+	++
gas1Δ	-	++	+
hog1Δ	+++	-	+++
fbp1Δ	++	+	++
fso1Δ	+++	++	+++
pro1Δ	++	+++	+++
veAΔ	+++	+	++
velBΔ	+++	+	++
tsc2Δ	+	+	-
ck1Δ	-	-	-

pass rate	cellophane penetration	mortality
+++; >80	+++; >1.5	+++; 0.8-1
++; 80-70	++; 0.5-1.5	++; 0.5-0.8
+; 45-70	+; 0.1-0.5	+; 0.2-0.5
-; <45	- ; <0.1	- ; <0.2

Movie Legends

Movie 1. *F. oxsporum* wt strain hyphae grown in the channels. Every 20 min, total 22 h, scale bar: 20 μm.

Movie 2. *F. oxsporum fmk1*Δ strain hyphae grown in the channels. Every 20 min, total 28 h, scale bar: 20 μm.

Movie 3. *F. oxsporum hog1* Δ strain hyphae grown in the channels. Every 20 min, total 12 h, scale bar: 20 μm.

Movie 4. *F. oxsporum mpk1* Δ strain hyphae grown in the channels. Every 20 min, total 27 and 30 h, scale bar: 20 μm.

Movie 5. *F. oxsporum chs5* Δ strain hyphae grown in the channels. Every 20 min, total 10 h, scale bar: 20 μm.

Movie 6. *F. oxsporum gas1* Δ strain hyphae grown in the channels. Every 20 min, total 28 h, scale bar: 20 μm.

Movie 7. *F. oxsporum tsc2* Δ strain (first half) and *ck1* Δ strain (second half) hyphae grown in the channels. Every 20 min, total 26 h, scale bar: 20 μm.

Movie 8. *F. oxsporum* wt strain hyphae grown in the channels imaged by holographic microscopy (upper) and DIC (lower). Every 80 min, total 8 h, scale bar: 20 μm.

Movie 9. *F. oxsporum mpk1* Δ strain hyphae grown in the channels imaged by holographic microscopy (upper) and DIC (lower). Every 80 min, total 12 h, scale bar: 20 μm.
